# Oxaluria—I

**Published:** 1908-09-26

**Authors:** 


### Medicine.

### OXALURIA—I.

It is a remarkable fact that oxalates, notwithstanding the ease with which they can be decomposed *in vitro*, undergo little or no oxidation in the body. It has been shown that nearly the whole of the oxalates that may be injected subcutaneously are recoverable from the urine unchanged.

There are two sources for the oxalates that are to be found in the urine, namely:—

1. *Exogenous oxalates*, taken into the patient's mouth ready formed in the food, particularly in vegetables. The best known example of a plant containing oxalic acid is sorrel—especially the wood-sorrel—often eaten raw for its refreshing acidity, but dangerous if consumed in any great quantity. It is from oxalis, the scientific name for wood-sorrel, that the acid was first named. Many vegetable foods contain definite quantities of oxalates, the following being a few examples:—

| | | | | | | |
|---|---|---|---|---|---|---|
| Cocoa | contains | 4.5 | grams | of oxalic | acid | per kilogram. |
| Tea | ,, | 3.7 | | ,, | ,, | ,, |
| Spinach | ,, | 3.2 | | ,, | ,, | ,, |
| Rhubarb | ,, | 2.4 | | ,, | ,, | ,, |
| Potato | ,, | 0.4 | | ,, | ,, | ,, |
| Cabbage | ,, | 0.2 | | ,, | ,, | ,, |
| Coffee | ,, | 0.1 | | ,, | ,, | ,, |

2. *Endogenous oxalates*, produced by metabolic processes within the body, either from proteid or from carbohydrate, possibly *via* glycuronic acid.

Very little is known about endogenous oxalic acid, and in the great majority of cases the factor which is responsible for variations in the quantity of oxalates in the urine is variability in the consumption of vegetables containing ready formed or exogenous oxalates.

In speaking of oxaluria, however, the clinician has acquired the habit of considering, not whether there is an absolute increase in the total quantity of oxalates in the urine, but merely whether there are or are not a greater number of crystals of oxalate of lime to be seen under the microscope than there should be. The estimation of the total quantity of oxalic acid in a urine is by no means easy, and at present such estimation is almost confined to scientific research work; it does not at present come into the sphere of practical medicine, for it has not yet been shown that any deductions of clinical value can be drawn from the figures when they have been obtained. Oxaluria from a clinical point of view, therefore, practically means the presence of excess of calcium oxalate crystals in the urine.

The questions which at once arise are: First, what causes oxalate crystals to appear in the urine at all? Secondly, what circumstances lead to these crystals being more numerous than usual? And thirdly, supposing the crystals are in excess of normal, that is to say, supposing there is oxaluria in the above restricted sense, what is its significance?

The possible oxalates in urine are: (1) those of the alkalies, potassium, sodium, and ammonium; (2) that of urea; (3) those of the alkaline earths, calcium and magnesium. The oxalates of sodium, potassium, and ammonium are all quite soluble, so that they never occur in urinary sediments or calculi. Oxalate of urea is also sufficiently soluble not to be found in urine under any but artificial circumstances; the addition of oxalic acid to a concentrated solution of urea will give a precipitate of urea oxalate; the crystals obtained are typical, and they have been used as a means of detecting urea, but under no ordinary conditions is the urine sufficiently concentrated, whilst at the same time containing sufficient oxalic acid to give any precipitate of oxalate of urea crystals. Oxalate of magnesium is also sufficiently soluble to remain dissolved in urine. The only salt of oxalic acid that occurs as a precipitate in urine is that in which the acid is combined with calcium. The sharply defined colourless octahedral crystals of $CaC_2O_4.3H_2O$, sometimes described as "envelope" crystals from their microscopical appearance as a square crossed both ways diagonally are to be mistaken for nothing else in urine.

Sometimes it is said that the shape of the calcium oxalate crystal is like a dumb-bell; but it is to be remembered that dumb-bell crystals in urine are very rare, that uric acid may also have the dumb-bell shape, and that either in the case of calcium oxalate or in that of uric acid the dumb-bell is not a true crystal but an imperfect one due to rapidity of formation. If oxalic acid is added to lime-water, the precipitate may contain many of these microscopic dumb-bells, but this is rarely the case with urine from which the crystallisation takes place slowly.

The separation of calcium oxalate depends upon many factors, amongst which may be noted:—

1. *The Dilution of the Urine.*—Oxalates may be precipitated even in dilute urines, but upon the whole they are more abundantly deposited in those that are concentrated.

2. *The Acidity of the Urine.*—They may come down in neutral or even in alkaline urines, but they come down most readily in acid, especially in highly acid urines.

3. *The Absolute Amount of Oxalic Acid Present.*—The facts that the sediment of one urine contains calcium oxalate crystals in abundance, and that that of another contains none, are no proof that the total quantity of oxalates differs in the two urines; it may even be that a urine with no calcium oxalate crystals in the deposit may contain a bigger total quantity of oxalic acid than one containing the crystals in abundance. Nevertheless, other things being equal, the bigger the total amount of oxalates in the urine, the greater the number of envelope crystals likely to be found.

4. *The Amount of Alkalies and Chlorides Present.* The effect of alkalies and chlorides upon oxalates is very complex; but the kind of way in which they act will be gathered from the fact that calcium oxalate is more soluble in aqueous solutions of chlorides of magnesium or calcium than it is in pure water.

5. *The Ratio of the Total Quantity of Calcium to the Total Quantity of Magnesium in the Urine.*—When calcium is relatively in excess the crystals come down; but when there is as much magnesium as calcium present the oxalic acid remains entirely in solution.

(*To be continued.*)

